# Loss of the Histone Pre-mRNA Processing Factor Stem-Loop Binding Protein in *Drosophila* Causes Genomic Instability and Impaired Cellular Proliferation

**DOI:** 10.1371/journal.pone.0008168

**Published:** 2009-12-04

**Authors:** Harmony R. Salzler, Jean M. Davidson, Nathan D. Montgomery, Robert J. Duronio

**Affiliations:** 1 Curriculum in Genetics and Molecular Biology, University of North Carolina, Chapel Hill, North Carolina, United States of America; 2 Department of Biology, University of North Carolina, Chapel Hill, North Carolina, United States of America; 3 Program in Molecular Biology and Biotechnology, University of North Carolina, Chapel Hill, North Carolina, United States of America; 4 Lineberger Cancer Center, University of North Carolina, Chapel Hill, North Carolina, United States of America; Texas A&M University, United States of America

## Abstract

**Background:**

Metazoan replication-dependent histone mRNAs terminate in a conserved stem-loop structure rather than a polyA tail. Formation of this unique mRNA 3′ end requires Stem-loop Binding Protein (SLBP), which directly binds histone pre-mRNA and stimulates 3′ end processing. The 3′ end stem-loop is necessary for all aspects of histone mRNA metabolism, including replication coupling, but its importance to organism fitness and genome maintenance in vivo have not been characterized.

**Methodology/Principal Findings:**

In *Drosophila*, disruption of the *Slbp* gene prevents normal histone pre-mRNA processing and causes histone pre-mRNAs to utilize the canonical 3′ end processing pathway, resulting in polyadenylated histone mRNAs that are no longer properly regulated. Here we show that *Slbp* mutants display genomic instability, including loss of heterozygosity (LOH), increased presence of chromosome breaks, tetraploidy, and changes in position effect variegation (PEV). During imaginal disc growth, *Slbp* mutant cells show defects in S phase and proliferate more slowly than control cells.

**Conclusions/Significance:**

These data are consistent with a model in which changing the 3′ end of histone mRNA disrupts normal replication-coupled histone mRNA biosynthesis and alters chromatin assembly, resulting in genomic instability, inhibition of cell proliferation, and impaired development.

## Introduction

Histones are a class of highly abundant nuclear proteins whose most basic function is to package and organize the genetic material. In addition to organizing DNA, histones play important roles in a number of other cellular processes critical for survival and development. These include DNA repair [1], chromosome segregation [2], regulation of transcription [3], and tissue differentiation [4]. In metazoans, there are two classes of histone proteins. The canonical, replication-dependent histones, H2a, H2b, H3, H4, and H1, are synthesized solely during S-phase, where they are utilized to package newly replicated DNA. The replication-independent histone variants are paralogs of the canonical histones which assemble into nucleosomes with specialized functions [5]. Unlike replication-dependent histones, histone variants can be synthesized and deposited into chromatin throughout the cell cycle [6].

Restriction of replication dependent histone biosynthesis to S-phase is conserved in all species of fungi, plants, and animals studied to date [7], [8]. Yeast and *Arabidopsis* accomplish S-phase coupling through transcriptional regulation [7]. In metazoans, S-phase coupled histone production is controlled largely through post-transcriptional regulation of mRNA levels due to changes in pre-mRNA processing efficiency and mRNA half life [7]. In budding yeast, the production of histones in the correct stoichiometry is important for genome maintenance and successful cell cycle progression [2], [9], [10], [11]. In human cells, the inhibition of histone gene expression leads to S-phase arrest [12], [13]. These observations demonstrate that proper regulation of replication-dependent histone production is functionally important.

Metazoan replication-dependent histone mRNAs are not polyadenylated [14], and instead terminate with a conserved 3′ stem-loop that is unique to histone mRNAs [15]. The regulatory properties conferred by the histone mRNA 3′ end are likely to impact the rate of histone protein synthesis, histone stoichiometry, and the timing of histone synthesis during the cell cycle [7], [8]. However, the precise connection between 3′ end mediated regulation of histone mRNAs and histone protein production *in vivo* remain to be determined. Changes in the way that the 3′ end affects mRNA processing, localization, or translation of histone mRNA could be reflected by changes in histone protein abundance or stoichiometry that alter properties of chromatin due to misregulation of chromatin assembly. In this study we test this hypothesis by analyzing mutations of the *Slbp* gene that disrupt normal histone mRNA 3′ end formation and regulation.

Formation of the histone mRNA 3′ end requires two unique sequence elements in the pre-mRNA. The first is the stem-loop, which remains part of the mature mRNA after pre-mRNA processing [16]. The stem-loop confers the specific coordinate regulation of replication-dependent histone mRNAs [17]. The second element is a purine rich histone downstream element (HDE), which is removed during the processing reaction [18]. Each of these sequences recruits factors that ultimately produce the single endonucleolytic cleavage between the stem-loop and HDE required for complete maturation of histone mRNAs [15].

The U7snRNP, composed of the U7 snRNA and a heptameric ring of Sm and Lsm proteins, is targeted to the HDE via base-pairing with the U7 snRNA [19]. Stem-loop binding protein (SLBP) directly binds the stem-loop of histone mRNAs and is necessary for correct processing of histone pre-mRNAs [20], and is absolutely required for processing *Drosophila* pre-mRNAs *in vitro* [21]. In addition, SLBP is required for the nuclear export and translation of histone mRNAs in mammalian cells [13], [22] . To date, there is no evidence for functions of SLBP outside those directly involved in histone biosynthesis. Additionally, other factors common to the canonical cleavage and polyadenylation reaction are required for histone pre-mRNA processing [23], [24], [25].

The maturation of histone mRNAs is a well-conserved process and has been characterized at the molecular level in considerable detail [15], [16]. Yet, despite the deep understanding of how the 3′ ends of histone mRNAs mature, the significance of this unique 3′ end in terms of replication-coupled histone protein production, genome integrity, and development have not been studied. This is due to difficulties in altering the 3′ ends of histone mRNAs in metazoans. Knocking out factors involved in processing mammalian histone mRNAs leads to a severe reduction in transcript levels [13], [26] making this approach unfruitful for specifically studying the significance of the 3′ end. A second difficulty is that replication-dependent histone genes typically occur in large clusters, making strategies that alter or replace the 3′ end *in vivo* problematic.

Investigation of this question in *Drosophila* provides a unique opportunity to overcome these difficulties. Unlike other organisms, the production of mature histone transcripts is not abrogated when normal processing of the pre-mRNA is prevented [27]. Null mutations in *Slbp* result in incorrectly processed histone mRNAs, due to read through and utilization of cryptic polyadenylation sites downstream of the normal cleavage site [27], [28], [29]. These transcripts contain both a stem-loop and a polyA tail and are not properly cell cycle regulated in some embryonic endocycling tissues, likely because they are not degraded at the end of S-phase [27], [28], [29].

*Drosophila Slbp* mutants support both DNA replication and chromatin assembly, and thus the aberrant histone mRNA in these mutants can be translated, though the rate and timing of translation is unknown. However, *Slbp* null mutants are developmentally delayed and die as pupae. [27], [28]. Because the histone mRNA 3′ end is thought to be involved in the coordinate regulation of replication-dependent histone production, and alterations in histone stoichiometry cause genomic instability [2], we considered the possibility that proper 3′ end-mediated regulation of histone mRNA might be important for genomic stability. Here, we demonstrate that *Slbp* mutants, despite sufficient histones to support DNA replication, display several forms of genomic instability and cell cycle abnormalities that disrupt development.

## Results

### Reduction of Slbp Causes Increased Frequency of Loss of Heterozygosity

**Figure 1:**
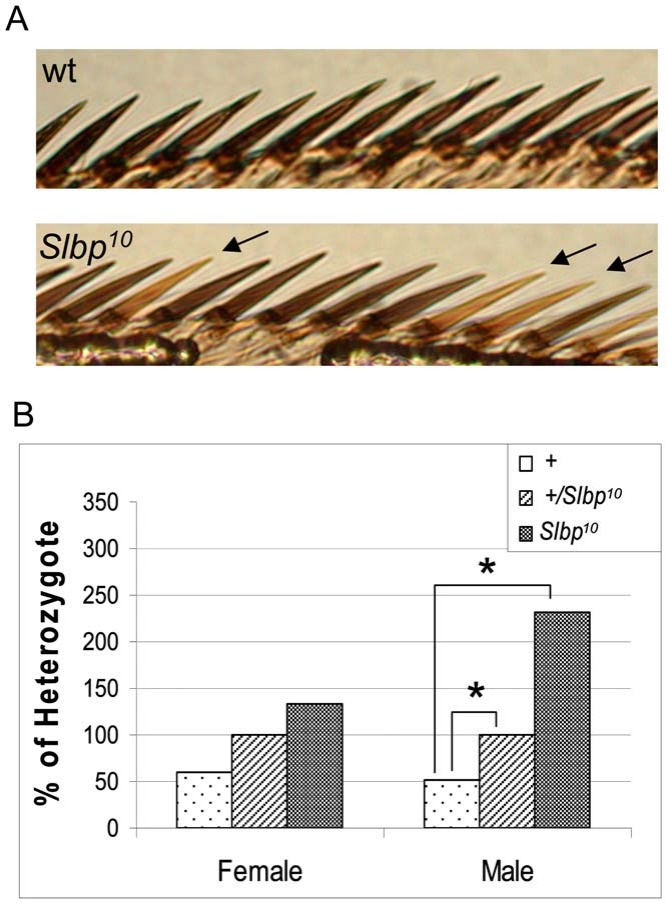
LOH is significantly increased in *Slbp* mutants. Wings from wt, *Slbp^10^* mutant flies heterozygous for a fourth chromosome translocation containing *y^+^* were mounted and the first twenty bristles of the anterior wing margin were used for analysis. A) Example of the data used for analysis. Yellow bristles indicate an LOH event (black arrows). B) For each *Slbp^10^* and* + *class, the frequencies of yellow bristles per wing were compared to heterozygous controls derived from a common culture. Frequency of yellow bristles was tabulated on a per wing basis. Data are expressed as a percentage of heterozygous controls, so that genotypes can be compared across experiments. 21-45 wings were analyzed per class. P-values are indicated as follows: * p<0.05, **p<0.01, ***p<0.001.

To gain a generalized measure of genomic instability, we developed a loss of heterozygosity (LOH) assay. Our assay measured loss of function of a *yellow^+^* (*y^+^*) locus, which is necessary for brown body pigment, from a 4^th^ chromosome translocation (the *y* ^+^ locus is normally located on the X chromosome). In flies heterozygous for this translocation, on an otherwise *y* genetic background, functional loss of the single *y^+^* locus on the translocation during development results in patches of yellow body color in adult flies, which are then scored as LOH events. To obtain a measure of LOH, we counted the frequency of yellow bristles found in the first twenty bristles of the anterior wing margin (Figure 1A). Since viable adults were required for this assay, we utilized the *Slbp^10^* hypomorphic allele. Wild type (wt) and mutant genotypes were normalized relative to *Slbp* heterozygous siblings. The heterozygotes present in every experiment controlled for variability in culture conditions, allowing comparison between experiments.

We analyzed varying doses of the *Slbp^10^* hypomorphic allele. In females, we observed a trend toward increased LOH with decreased dosage of *Slbp* (Figure 1B). In males, we found significant differences between wt and the *Slbp^10^/+* heterozygous normalizing group, as well as between *Slbp^10^* homozygous mutants and heterozygous controls. The frequency of LOH in *Slbp^10^/+* heterozygous males was ∼1.9 fold that of wt siblings (p<0.012), while *Slbp^10^* homozygous mutants had ∼2.3 fold the LOH of heterozygotes (p<0.011) (Figure 1B). These results demonstrate that decreasing Slbp function increases LOH.

Interestingly, we observed that the LOH phenotype was more severe in males. One possible explanation for this result might be the presence of the highly heterochromatic Y chromosome. This heterochromatic region of the genome sequesters histones, and has been shown to affect chromatin related phenotypes in other assays [30], [31], [32].

### *Slbp* Mutants Exhibit Increased Frequency of Double Strand Breaks and Tetraploid Cells

Functional LOH can be caused by a number of processes including spontaneous mutation, mitotic crossovers events, chromosome loss, chromosome breakage, and heterochromatin spreading that reduces gene expression.

In order to determine which of these processes might contribute to the LOH we observed in *Slbp* mutants, we examined metaphase chromosome preparations of *Slbp^15^* null mutant larval brain neuroblasts. The karyotype of larval brain neuroblasts can be easily analyzed for chromosomal abnormalities, such as breaks and changes in ploidy.

**Figure 2:**
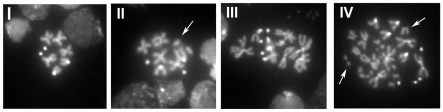
*Slbp* mutants have increased DNA damage and exhibit tetraploid nuclei. Larval neuroblast karyotypes were obtained for *Slbp^15^* and *w^1118^* 3^rd^ instar larvae. Each karyotype was assigned to one of four categories: I) Normal karyotype with no chromosomal breaks II) Normal karyotype with at least one chromosomal break (arrow), III) Tetraploid with no breaks, IV) Tetraploid with at least one break (arrows). White arrows indicate examples of chromosomal breaks.

First, we quantified the frequency of chromosome breaks. A normal *Drosophila* karyotype contains three pairs of autosomes and one pair of sex chromosomes (Figure 2, category I). Nuclei were scored positive for breaks if there was at least one broken chromosome arm, relative to a normal karyotype, regardless of the extent of damage (Figure 2, category II, IV). The baseline frequency of *wt* nuclei containing breaks was 2.46% in this assay (Table 1, II + IV), which is slightly higher than, but comparable to, previous reports [33]. Cells containing more than a single break were extremely rare. In contrast, we observed a significant increase in chromosome breaks in the *Slbp^15^* (p<0.03) mutant, with 8.43% of nuclei containing breaks (Table 1, II + IV). In the mutant genotype, cells containing multiple breaks or other types of chromosomal abnormalities occurred at much greater frequency. The increase in chromosomal breaks in *Slbp^15^* mutants likely contributes to the increased LOH observed in these mutants.

**Table 1:** Mean Percentage of Mitotic Nuclei Per Brain in Each Class.

	*wt*	*Slbp^15^*
I	97.5±0.86	87.2±2.36
II	2.46±0.86	5.52±1.55
II + IV	2.46±0.86	8.43±1.89
III	0	4.37±1.16
III + IV	0	7.29±2.26
IV	0	2.91±1.77

For each genotype, the percentage of nuclei containing chromosomal breaks and tetraploidy was calculated for 6 individual brains and mean percentages determined. 23–132 mitotic nuclei per individual brain were analyzed. Four classes of abnormal karyotypes (see Figure 2): I) Normal karyotype with no breaks. II) Diploid nuclei containing breaks. II + IV) All nuclei containing breaks. III) Tetraploid nuclei without breaks. III + IV) Total tetraploid nuclei including those with breaks. IV) Tetraploid nuclei with breaks.

We next measured ploidy in *Slbp^15^* mutants. The frequency of cells containing one or more extra chromosomes was quantified for *wt* and *Slbp^15^* genotypes. We chose to determine polysomy rather than monosomy because it is impossible to distinguish true monosomy from loss of chromosomes due to preparation artifacts. Each normal mitotic nucleus possesses eight sister chromatid pairs (Figure 2, category I). We found that *Slbp^15^* mutants were not statistically different from wt controls in percentage of polysomic cells (data not shown). Thus, it is unlikely that increased aneuploidy can account for the dramatic increase in LOH in *Slbp* mutants in our previous analysis. Consistent with this assertion, yellow bristles scored in the LOH assay did not have a Minute phenotype, which would result from loss of the entire translocated 4^th^ chromosome containing *y^+^* [34].

Unexpectedly, we detected the presence of tetraploid cells in *Slbp^15^* mutant brains (Figure 2, category III). Tetraploidy can arise from several mitotic defects including failure of cytokinesis and prolonged arrest at the spindle checkpoint due to DNA damage, followed by subsequent cell cycle reentry [35]. Whereas tetraploidy was never detected in wild type cells, we observed an average of 7.29% tetraploid nuclei per brain in *Slbp^15^* mutants (p<0.03) (Table 1, III + IV). Furthermore, a disproportionately large proportion of tetraploid cells also contained chromosomal breaks (Table 1, category IV). This is consistent with tetraploidy in *Slbp* mutants being caused by prolonged arrest at the spindle checkpoint due to damage to the genome.

### *Slbp* Mutants Exhibit Enhancement of Position-Effect Variegation

Silencing of a locus by surrounding heterochromatin could result in a functional LOH, even though the locus itself may be intact. Changes in histone gene dosage can alter the extent of heterochromatin [36], [37]. Alternatively, mutations which promote relaxation of chromatin structure could also indirectly increase LOH by leaving the chromosome more susceptible to breaks. We reasoned that altered chromatin structure resulting from the misregulation of histone biosynthesis might account for some of the genomic instability we observed in *Slbp* mutants. Therefore, we hypothesized that changes in chromatin structure would correspond with the observed increases in genomic instability.

To analyze changes in chromatin structure, we measured position-effect variegation (PEV) in *Slbp* mutants. PEV is a phenomenon whereby gene expression is modulated by the structure of surrounding chromatin. Surrounding heterochromatin can spread into a normally transcriptionally active gene, silencing expression. In PEV, the expressed or silent state of gene expression is propagated clonally, producing a variegated phenotype. The extent of variegation can be modified when genes involved in the formation of chromatin structure are mutated. Mutations that inhibit heterochromatin formation suppress variegation, whereas mutations that increase the abundance of heterochromatin enhance variegation.

To measure PEV in *Slbp* mutants, we observed the extent of variegation of the *w^m4^* inversion. The *w^m4^* inversion is an X-chromosome inversion that relocates the *w^+^* gene from the tip of the X chromosome to a region proximal to the pericentric heterochromatin. Expression of the *w^+^* gene, which is necessary for pigment deposition in the eye, is modulated clonally by the state of this nearby chromatin. This enables both eye pigment variegation and total amount of pigment accumulation to be used as read-outs of chromatin structure.

**Figure 3:**
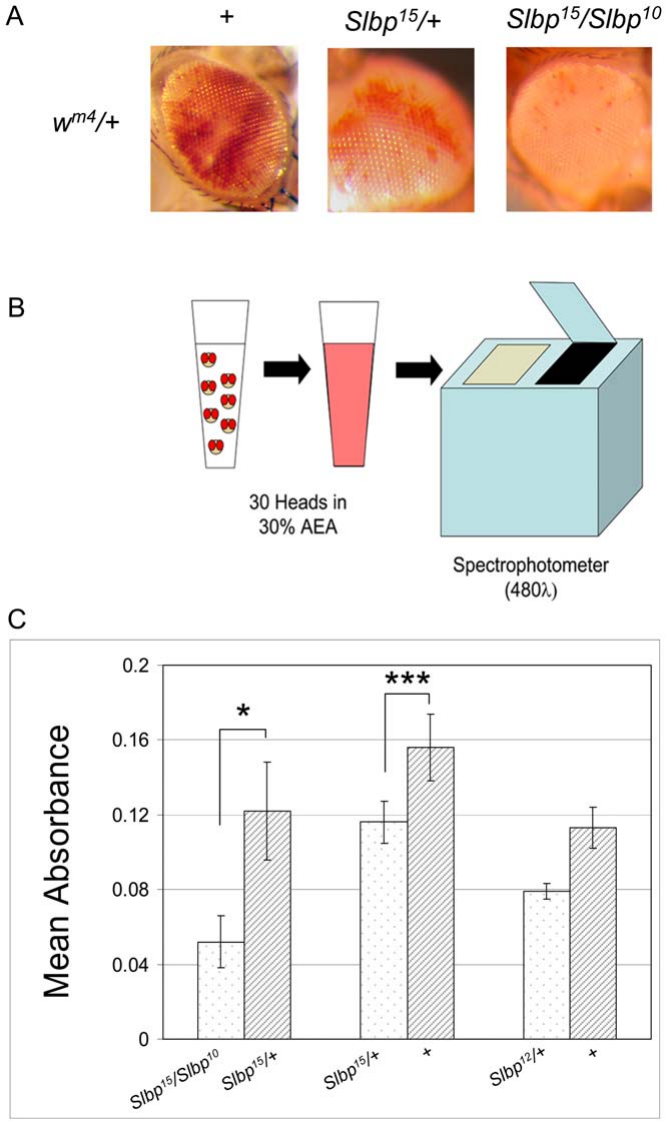
*Slbp* mutants are modifiers of PEV. Eyes were analyzed for extent of variegation in flies carrying one copy of the *w^m4^* inversion with one of the following genotypes: *Slbp^15^/Slbp^10^, Slbp^15^/+,* and *wt*. A) Representative eyes from females of each genotype. B) Quantitative assay for PEV. Eye pigment was quantified by measuring absorbance at 480λ for 30 fly heads per sample. C) Absorbance values of samples from allelic combinations of *Slbp* mutations. For each genotype, n = 10, except when comparing *Slbp^15^/Slbp^10^* and *Slbp^15^/+*, for which n = 3. Each pair of bars represents a single experiment. P-values are indicated as follows: * p<0.05, **p<0.01, ***p<0.001. Error bars indicate SEM.

We constructed a series of *Slbp* genotypes in conjunction with one copy of the *w^m4^* inversion. By inspection of adult eyes, we observed that pigment expression decreased with decreasing dosage of *Slbp* (Figure 3A). This indicates that *Slbp* mutation is an enhancer of PEV. To confirm this quantitatively, we utilized a spectrophotometer to measure the absorbance of eye pigment from samples of each genotype [38] (Figure 3B). The absorbance of each sample was compared to that obtained from siblings from the same culture vial to control for parental and culture dependent effects.

We quantified pigment levels in *Slbp* mutants. *Slbp^15^/Slbp^10^* is the allelic combination with the smallest dose of SLBP compatible with adult viability. Pigment absorbance values in *Slbp^15^/+* heterozygous sisters were ∼2.3 fold greater than those of *Slbp^15^/Slbp^10^* females (Figure 3C, left) (p<0.04). The *Slbp^15^* mutation also acts as a dominant enhancer of PEV since the wt absorbance was ∼1.4 fold greater than that of *Slbp^15^* heterozygotes (Figure 3C, middle) (p<0.0001). To ensure that these PEV phenotypes were due to the *Slbp^15^* mutation and not a component of the genetic background, we also examined the *Slbp^12^* null mutation, which was derived from a different P-element insertion [27]. We observed that *Slbp^12^*/+ females also exhibited PEV enhancement with wt pigment levels being ∼1.4 fold greater than that of the heterozygotes (Figure 3C, right) (p<0.0001). We were unable to analyze males quantitatively because *Slbp* mutation enhanced variegation so much that pigment could not be accurately measured.

These data demonstrate that *Slbp* mutation enhances PEV of the *w^m4^* inversion, suggesting that *Slbp* mutation augments heterochromatin formation. Our data show that mutation of a factor required for proper processing and post-transcriptional regulation of histone mRNAs alters chromatin structure. Furthermore, we propose that changes in chromatin structure in *Slbp* mutants contribute to overall genomic instability.

### The Steady State Level of Replication-Dependent Histones Does Not Change in *Slbp* Mutants

**Figure 4:**
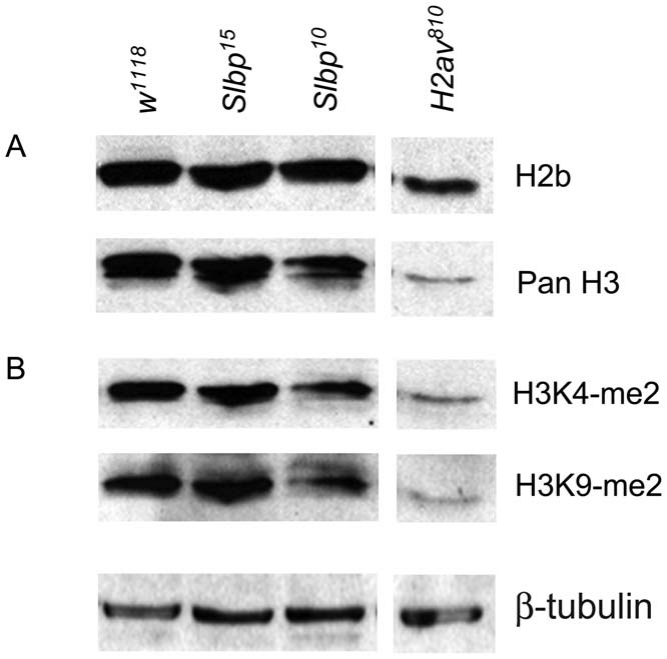
*Slbp* mutants have no detectable global change in histone protein levels. Protein lysates from *wt, Slbp^15^, Slbp^10^*, and *H2aV^810^* mutants were obtained from whole 3^rd^ instar larvae and probed with antibodies to A) H2b and H3, and B) H3K4-me2 and H3K9-me2. β-tubulin was used as a loading control for both panels.

Alterations in histone gene copy number and histone protein levels result in changes in chromatin structure and genome stability [2], [10], [37]. Given the known involvement of Slbp in histone mRNA biogenesis, as well as our observations that chromatin structure and genome stability are compromised in *Slbp* mutants, we determined whether the total amount of histone protein in *Slbp* mutants is different than wt. To do this, we measured histone H2b and H3 levels by western blot analysis of whole 3^rd^ instar larval extracts from homozygous *Slbp^15^* and *Slbp^10^* mutants, and compared the amount of histones to wild type counterparts in at least five separate experiments. We found no substantial or consistent change in level of histones H2b and H3 in *Slbp^15^* or *Slbp^10^* mutants (Figure 4A). As an indication that we could detect a difference in the amount of core histone protein, we consistently observed a decrease in H2b and H3 in *H2aV^810^* mutant larvae. *H2aV* encodes a histone variant that when mutated causes misprocessing of histone mRNAs [25] and suppresses PEV [39]. We also measured steady state H2a and H3 abundance in histone extracts prepared from isolated nuclei, and again found no effect due to *Slbp* mutation (data not shown). These results indicate that the bulk amount of histone proteins present in *Slbp^10^*and *Slbp^15^* null mutants is similar to wild type. We cannot rule out either very subtle alterations in protein levels undetectable by our western blot assay or tissue specific differences in histone protein production. However, we conclude that the genomic instability and modification of PEV that we observed in *Slbp* mutants cannot be explained by large changes in the total amount of replication-dependent histone proteins

### Distribution and Abundance of Euchromatic and Heterochromatic Markers Do Not Change in *Slbp* Mutants

Our results demonstrate that *Slbp* mutants exhibit genomic instability and changes in the extent of pericentric heterochromatin, but not detectable changes in the overall amount of histone protein. An alternative explanation for the observed genomic instability is that differences in timing of histone production during the cell cycle rather than the amount of histone synthesis leads to abnormal chromatin assembly. We previously showed that the misprocessed, polyadenylated histone mRNAs produced in *Slbp* mutants are not always properly cell cycle regulated and in some tissues accumulate in cells that are not synthesizing DNA [27], [29]. Consequently, this misregulation could lead to production of replication-dependent histone proteins outside of S-phase, perhaps interfering with deposition of replication-independent histone variants. Two of these variants, H2aV and Cid, have roles in establishing various types of heterochromatin, while H3.3 is enriched in euchromatin [5]. This led us to hypothesize that the global euchromatin/heterochromatin balance would be shifted in histone pre-mRNA processing mutants.

To measure this, Western blots of whole 3^rd^ instar larval extracts were probed for euchromatic and heterochromatic markers. We chose dimethylation of H3-K4, a histone modification associated with transcriptionally active genes, as a marker of euchromatin [40], [41]. H3-K9 dimethylation (H3K9-me2) was used as a marker for heterochromatin [42]. We observed no reproducible difference in the ratio of H3K4me2 to H3K9me2 between *Slbp^15^* and *Slbp^10^* mutant third instar larval protein extracts and wild type controls (Figure 4B). Furthermore, we detected no substantial difference in any of our mutants when we considered either marker relative to a loading control (Figure 4B).

**Figure 5:**
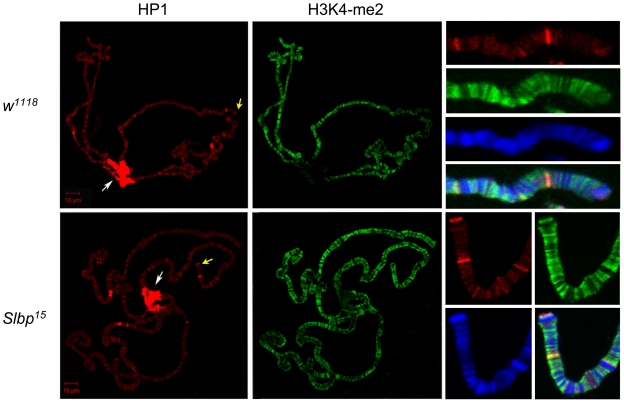
The global balance of euchromatin and heterochromatin remains unchanged in *Slbp* mutants. Polytene chromosome spreads of *wt* and *Slbp^15^* mutants were stained with antibodies for HP1 (red) and H3K4-me2 (green). DNA was stained with DAPI (blue). Leftmost panels are low magnification images of the entire genome. HP1 stains the chromocenter (white arrows) and telomeres, as well as other heterochromatic bands. Rightmost panels are high magnification images of a single homologous stretch of autosome for each genotype. Position of high magnification images are indicated on low magnification images with yellow arrows.

It is possible that cell type specific changes in chromatin structure might be masked by assessing H3 methylation status in the whole animal. We therefore decided to ascertain the distribution of euchromatic and heterochromatic markers in a specific tissue, the larval salivary gland. This tissue was chosen because the large polytene chromosomes allow exceptional spatial resolution, and the wild type distribution of certain euchromatic and heterochromatic markers is well characterized. We again chose H3K4-me2 as our euchromatic marker. On polytene chromosomes, this modification is localized to euchromatic interbands which contain most active genes, and is mostly excluded from the chromocenter and other heterochromatic regions [43]. Heterochromatin protein 1 (HP1), a well-studied heterochromatin structural protein, was chosen as a marker of heterochromatin [44]. On polytene chromosomes, HP1 localizes to the chromocenter, telomeres, transposon arrays, and various heterochromatic bands (Figure 5) [45].

Polytene chromosome spreads of *Slbp^15^* mutant larvae were immunostained for H3K4-me2 and HP1. In wild type larvae, patterns of H3K4-me2 & HP1 distribution recapitulated previously published studies [43]. However, we observed no gross differences in the distribution or abundance of either marker in *Slbp^15^* mutant salivary gland chromosomes (Figure 5). These data support the conclusion that the global distribution of euchromatin and heterochromatin in *Slbp* mutants is largely unaltered in larval endocycling tissues. However, this does not exclude the possibility that the distribution of euchromatin and heterochromatin might be altered in diploid tissues, such as brain and imaginal discs, as suggested by the PEV assay.

### *Slbp* Mutants Exhibit Proliferation Defects and a Prolonged S-Phase

DNA replication and histone synthesis are coupled [12], [46]. One advantage of this coupling might be to prevent DNA damage during S phase. Slowed assembly of chromatin behind the replication fork can result in collapsed forks [47], which can be processed into double strand breaks, causing genomic instability [47], [48], [49]. Mutations that prolong S-phase in *Drosophila*, such as in the *Orc2, Orc5, PCNA, and Mcm4* genes, produce defects observable in mitotic chromosome spreads, including chromatid breaks [50], [51]. If the special 3′ end of histone mRNAs is needed for coupling rates of histone protein synthesis with DNA replication, then *Slbp* mutant cells may have chromatin assembly defects that cause prolonged S-phase, resulting in DNA damage and impaired cell proliferation.

**Figure 6:**
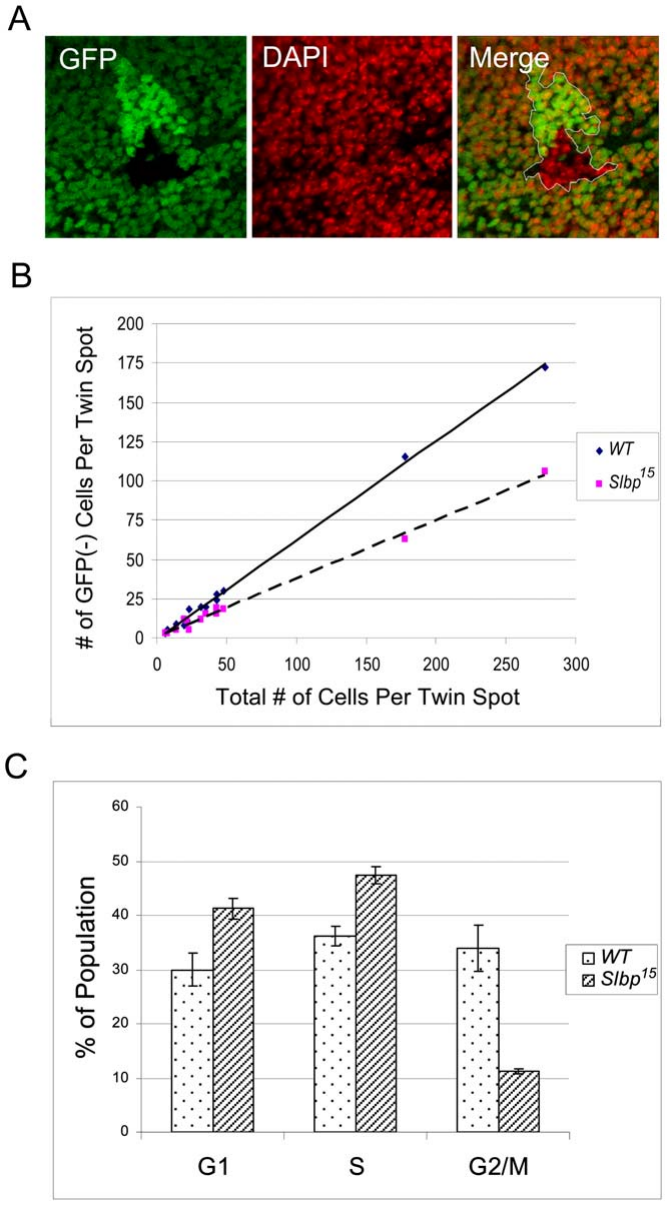
*Slbp* mutants exhibit a cell proliferation defect. A) Mitotic clones from *Slbp^15^/Ubi:GFP-nls* 3^rd^ instar larvae are visualized with native GFP signal and stained with DAPI. B) Cell counts of *wt* or *Slbp^15^* mutant GFP negative cells plotted against the total number of cells in each twin spot with accompanying trend line for each genotype. A solid line indicates *wt*, dashed indicates *Slbp^15^*. For the number of twin spots analyzed, (n = 13) P-values are indicated as follows: * p<0.05 and n.s. = not significant. C) FACS analysis of *wt* and *Slbp^15^* mutant wing discs from wandering 3^rd^ instar larvae. Each bar represents the mean of three independent experiments where over 4,000 cells were analyzed per genotype. P-values are indicated as follows: ***p<0.001. Error bars indicate SEM.

To test whether *Slbp* mutants exhibit a proliferation defect, we performed a “twin spot” analysis in wing imaginal discs by inducing mitotic clones using FLP recombinase [52]. Induction of mitotic recombination in larvae heterozygous for the *Slbp^15^* mutation and a GFP expressing transgene produced a characteristic twin spot derived from the proliferation of wt and *Slbp^15^* mutant daughter cells (Figure 6A). A range of clone sizes was produced, corresponding to the timing of each mitotic recombination event (Figure 6B). Cell proliferation was assessed by comparing the number of *Slbp^15^* mutant to the total number of cells in each twin spot. The number of cells was determined using the two confocal images from a Z-stack that contained the largest two-dimensional area. To get an estimate of relative proliferation rate, we plotted both mutant and wt cell numbers vs. the total number of cells for each twin spot and fitted a trend line to each set of points (Figures 6B). This analysis revealed a significant proliferation defect in *Slbp^15^* mutant imaginal disc cells (p<0.04) (Figure 6B). Furthermore, this defect is more pronounced in larger clones, most likely because SLBP protein becomes increasingly depleted during each successive cell division. These results are consistent with the observation that *Slbp^15^* mutant larvae exhibit a developmental delay before the onset of lethality.

If DNA damage and genome instability are caused by impaired chromatin assembly during DNA replication, we would predict that progression of cells through S-phase would be slowed relative to other phases of the cell cycle. To assess whether the proliferation defect observed in *Slbp* mutant cells corresponds with impaired progression through S-phase, we utilized FACS to profile cell cycle phasing in wing imaginal disc cells of 3^rd^ instar wt and *Slbp^15^* mutant larvae (Figure 6C). Using data from three separate experiments, we observed that cell cycle phasing in *Slbp^15^* wing disc cells was significantly different than wt (p<0.0001). In *Slbp^15^* mutant wing discs, 41.3% of cells were in G1, compared with 30.0% in wt discs. Similarly, 47.7% of *Slbp^15^* mutant cells were in S-phase, compared with only 36.2% in wt discs. Conversely, we observed relatively few cells with G2 DNA content in *Slbp^15^* mutant wing discs (11.3%), compared with wt (33.9%) (Figure 6C). Because it is difficult to distinguish cells in G1 from very early S-phase by FACS, the increased G1 population in *Slbp* mutants may represent cells that are progressing very slowly through early S-phase. These data demonstrate that *Slbp^15^* mutant cells have a cell cycle defect, and are consistent with a model where impaired chromatin assembly capability impedes S-phase progression, producing DNA damage, genomic instability, and delayed mitosis.

## Discussion

In this report we identified four forms of genomic instability in *Drosophila Slbp* mutants: an increase in loss of heterozygosity, localized changes in heterochromatin structure as measured by modification of PEV, tetraploidy, and chromosomal breaks. Some of these measurable genomic defects may cause the lethality of *Slbp* null mutations. A large body of prior work indicates that SLBP participates in the processing of histone pre-mRNA resulting in formation of a unique mRNA 3′ end that is not polyadenlylated [7], [8]. In *Drosophila*, Slbp is essential for histone pre-mRNA processing, and all replication-dependent histone mRNAs are instead converted into polyadenylated mRNAs [27], [29]. There is no evidence from genome wide studies that Slbp is capable of binding non-histone RNAs [53] and SLBP has never been implicated in the direct regulation of any genes other than the histone genes. Thus, the simplest interpretation of our data is that proper histone mRNA 3′ end formation and its accompanying regulation are necessary for accurate replication and propagation of the genome. Changes in chromatin assembly and structure in *Slbp* mutants might generally increase the susceptibility of the genome to damage.

Some parameters that we anticipated might be causal for these genomic instability phenotypes are relatively normal. We observed no changes in total histone protein levels, and no overt, global changes in chromatin structure as measured cytologically in polytene chromosomes using antibodies that recognize a histone modification enriched in euchromatin or heterochromatin. One possibility is that perturbations in the rate of histone protein expression during S phase or timing of expression relative to the cell cycle, rather than to changes in absolute amounts of histone protein, causes the genomic instability in *Slbp* mutants.

This possibility is consistent with our observations of impaired proliferation of *Slbp* mutant wing imaginal disc cells. Reduced histone protein synthesis may be compensated by slowing down the cell cycle, which would result in no observed change in histone protein abundance. In addition, reduced proliferation and slow organismal growth of *Slbp* mutants could be a direct consequence of impaired chromatin assembly in S-phase, which produces S-phase arrest and double-strand breaks in human cell lines [49]. In fact, work in mammalian cell culture demonstrates that the stem-loop on histone mRNAs is necessary for coupling histone mRNA stability to DNA synthesis during S-phase by way of the DNA-damage responsive ATR pathway [54], [55]. Any change in rate of histone protein synthesis producing a commensurate slow-down in cell proliferation might result in little or no change in assays which measure a snapshot of a dynamic state, such as global chromatin structure or total histone protein abundance.

Our data also suggest that slowed progression through S-phase contributes to the proliferation defect observed in *Slbp* mutants. This is similar to observations that RNAi knockdown of Slbp in mammalian cells results in delayed progression through S-phase [56]. Unlike *Drosophila* cells, mammalian cells depleted of SLBP produce very little polyadenylated histone mRNA, and instead fail to accumulate and export normal amounts of histone mRNA [13]. Thus, the common phenotype of S-phase delay may result from impaired production of histone protein, which may be caused by inefficient translation of polyA histone mRNA in the absence of SLBP, which is known to stimulate histone mRNA translation in vertebrates [57], [58].

In conclusion, our data suggest that proper histone mRNA 3′ end formation is necessary for maintaining genomic stability and normal cell cycle progression. We propose a model in which inefficient chromatin assembly during S-phase in histone mRNA processing mutants causes DNA damage, genomic instability, and problems with cell proliferation, leading to impaired development.

## Materials and Methods

### Fly Strains

*Slbp^10^, Slbp^12^, Slbp^15^* [27], *H2av^810^* [59], *w^m4^* [60], *C(1;Y)1, y^1^/y^1^ f^1^; Dp(1;Y;4)y^+^, sv^spa-pol^* [*61*] and *Df(3R)3450* [62] were characterized previously. Stocks used for clonal analysis and the *w^1118^* strain were obtained from Bloomington Stock Center.

### Loss of Heterozygosity (LOH) Analysis

Flies of the following genotypes were used for LOH analysis: *y;Slbp^10^; T(1;4)y^+^, y;Slbp^10^/TM3, Sb; T(1;4)y^+^, y;+/TM3, Sb; T(1;4)y^+^*. Wings were dissected and the first 20 bristles of the anterior wing margin were imaged on a standard light microscope. The number of yellow bristles was counted from each image. Statistical analysis was conducted using InStat (Graph Pad), comparing numbers of yellow bristles per wing among classes. Two-tailed p-values from the non-parametric Mann-Whitney test are reported.

### Metaphase Spreads

Metaphase spreads were conducted as described [63]. Briefly, brains from wandering 3^rd^ instar larvae were dissected in saline. Swelling was induced with 0.5% sodium citrate. Neuroblasts were fixed for 10 seconds in a solution of 46% acetic acid, 46% methanol, and 8% ddH_2_O. Brains were then incubated in 45% acetic acid and squashed under a siliconized coverslip, dehydrated in 95% EtOH, rehydrated in 2X SSC, and stained with 0.1mg/mL DAPI. In experiments assessing chromosomal abnormalities, brains were incubated for 90 minutes in 0.1 mM colchicine to induce mitotic arrest prior to sodium citrate treatment. Individual nuclei were imaged and the karyotype was ascertained. For each brain, the percentage of nuclei in each category was calculated. Microsoft Excel was used to perform unpaired *t*-tests between groups.

### PEV Assay

Flies of the following genotypes were utilized for analysis: *In(1)w^m4^/w;Slbp^10^/Slbp^15^, In(1)w^m4^/w; Slbp^15^/TM3, Sb, In(1)w^m4^/w; +/TM3, Sb*. Pigmentation was quantified as described [38]. Briefly, newly eclosed flies were collected and aged for four days. Flies were decapitated and pigment from 30 heads per sample was extracted in 1 ml of 30% Acidified Ethyl Alcohol (AEA) over the course of 3 days. Absorbance readings were obtained at 480 nm on an Eppendorf spectrophotometer. P-values from two-tailed paired *t*-tests were ascertained using InStat (Graph Pad). For each experiment, 10 sample pairs were compared except when comparing *Slbp^15^/Slbp^10^* and *Slbp^15^/+* flies. These transheterozygotes did not eclose at a Mendelian frequency, thus only 3 sample pairs were analyzed.

### Immunoblots

Protein was obtained from 50 wandering 3^rd^ instar larvae by grinding with a Polytron homogenizer in 1 ml NET buffer + Protease inhibitors (50 mM Tris, 400 mM NaCl, 5 mM EDTA, and 1% NP40+1.5 ug/mL aprotinin, 0.7 ug/ml pepstatin A, 0.5 ug/ml leupeptin, 1 mM PMSF) and 50 mM Sodium Butyrate. 50–100 mg of protein was loaded onto a 15% Tris HCl gel (BioRad) and transferred to a PVDF membrane with 0.2 um pore size. Blots were blocked in 5% milk in PBS-T and incubated in primary antibody overnight at 4°. Polyclonal rabbit C-terminal H3 (1∶3000) (Abcam #1791), polyclonal rabbit H2b (Abcam #1790) (1∶3000), monoclonal mouse H3K9-me2 (Abcam #1220) (1∶750), and polyclonal rabbit H3K4-me2 (Abcam #7766) (1∶3000), and monoclonal mouse Β-tubulin (1∶1000) antibodies were obtained commercially. Blots were washed in PBS-T and incubated for 1 hr at RT in either αRabbit-HRP (Amersham) (1∶1000) or αMouse-HRP (Amersham) (1∶1000). Presence of antibody was ascertained either with ECL or ECL+ (Amersham).

### Polytene Chromsome Spreads

Polytene squashes were prepared as described [64] with the following modifications. Instead of moving glands between solutions, solutions were exchanged on the coverslip. Incubation time in 3.7% paraformaldehyde, 0.1% Triton-X in PBS was extended to 2 minutes. Squashes were incubated in primary antibodies overnight at 25° unless otherwise noted. Primary antibodies used include monoclonal mouse H3K9-me2 (Abcam #1220) (1∶100), polyclonal rabbit H3K4-me2 (Abcam #7766) (1∶500), monoclonal mouse C1A9 for HP1 (Developmental Studies Hybridoma Bank) (1∶100). Slides were washed in PBS-T and incubated for 1 hr at RT in either αRabbit-cy5 (Jackson) (1∶500) or αMouse-cy3 (Jackson)(1∶500), and then stained with DAPI. Chromosomes were imaged in stacks on a Zeiss 510 confocal microscope.

### Clonal Analysis

Clones were generated using the Flp recombinase system described previously [52]. First and second instar larvae were heat shocked for 35 minutes. Wing discs were fixed 3-4 days after heat shock in 4% paraformaldehyde for 20 minutes and stained for Armadillo (Developmental Studies Hybridoma Bank) (1∶50), as well as DAPI. Clones were imaged on a Zeiss 510 confocal microscope, and cell counts were taken from the two slices which represented the largest total area. P-values for twin spot analysis were obtained using a two tailed paired *t*-test.

### FACS Analysis

FACS analysis on wing discs was performed as described previously [65], [66]. Briefly, wandering 3rd instar wing imaginal discs were dissected in PBS during a stage matched one hour developmental window. Discs were dissociated with 10X trypsin-EDTA (Sigma) and DNA was labeled with 1X Hoescht 33342 (Acros Organics) in 1X PBS for 3 hours rocking. Flow cytometry was performed using a LSR II (BD), and the data was analyzed with FloJo version 7.2.5 software (FloJo). Percentages of G1, S, and G2 were calculated using the ModFit LTTM software (Verity Software House). P-values were obtained using a χ^2^ independence test.
